# Supplementary material for: Host Genetics and the Skin Microbiome Independently Predict Parasite Resistance
Source: Ecol Evol. 2026 Jan 21;16(1):e72923. doi: 10.1002/ece3.72923 (PMC12822735; doi:10.1002/ece3.72923)
Supplement: Supplementary file 2 — Appendix S2: ece372923‐sup‐0002‐AppendixS2.pdf. [file ECE3-16-e72923-s001.pdf]

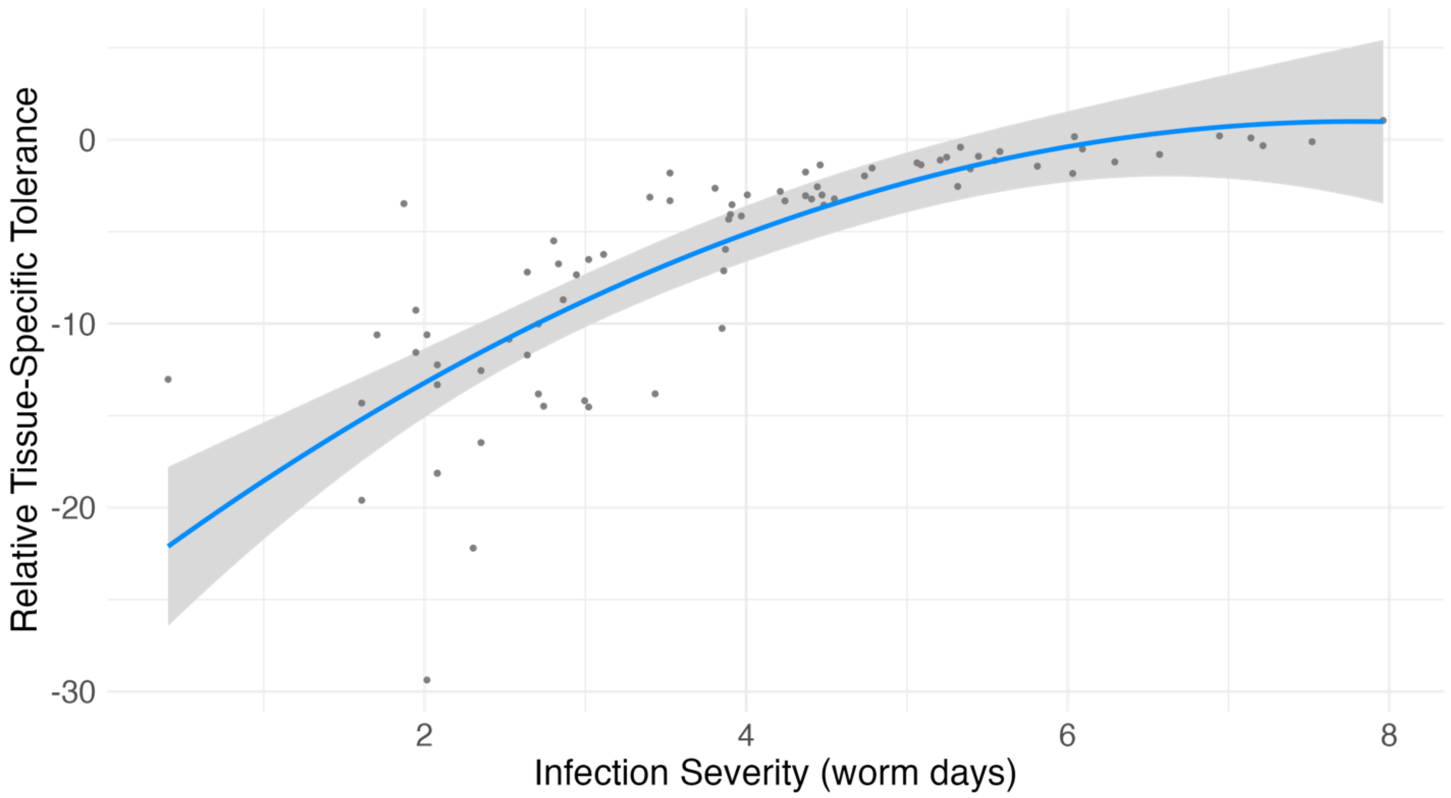

Figure S1. Polynomial regression demonstrates a trade-off between relative tissue-specific tolerance and parasite resistance (the inverse of infection severity). Points are back-transformed partial residuals from the model, and curves and shading give the model fits and 95% confidence bands.

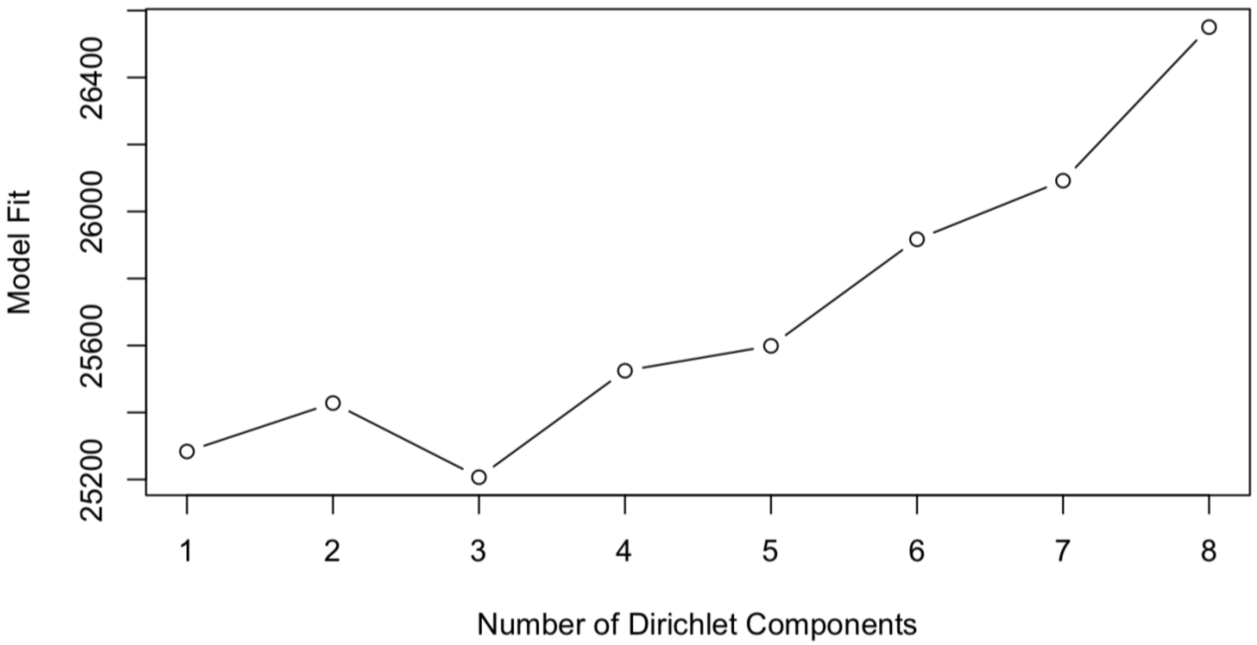

Figure S2. Identifying number of community types in pre-infection skin swabs of Guppies (n=50). Model fit (LaPlace approximation) with the number of Dirichlet components (k=3) was the lowest and best fit.

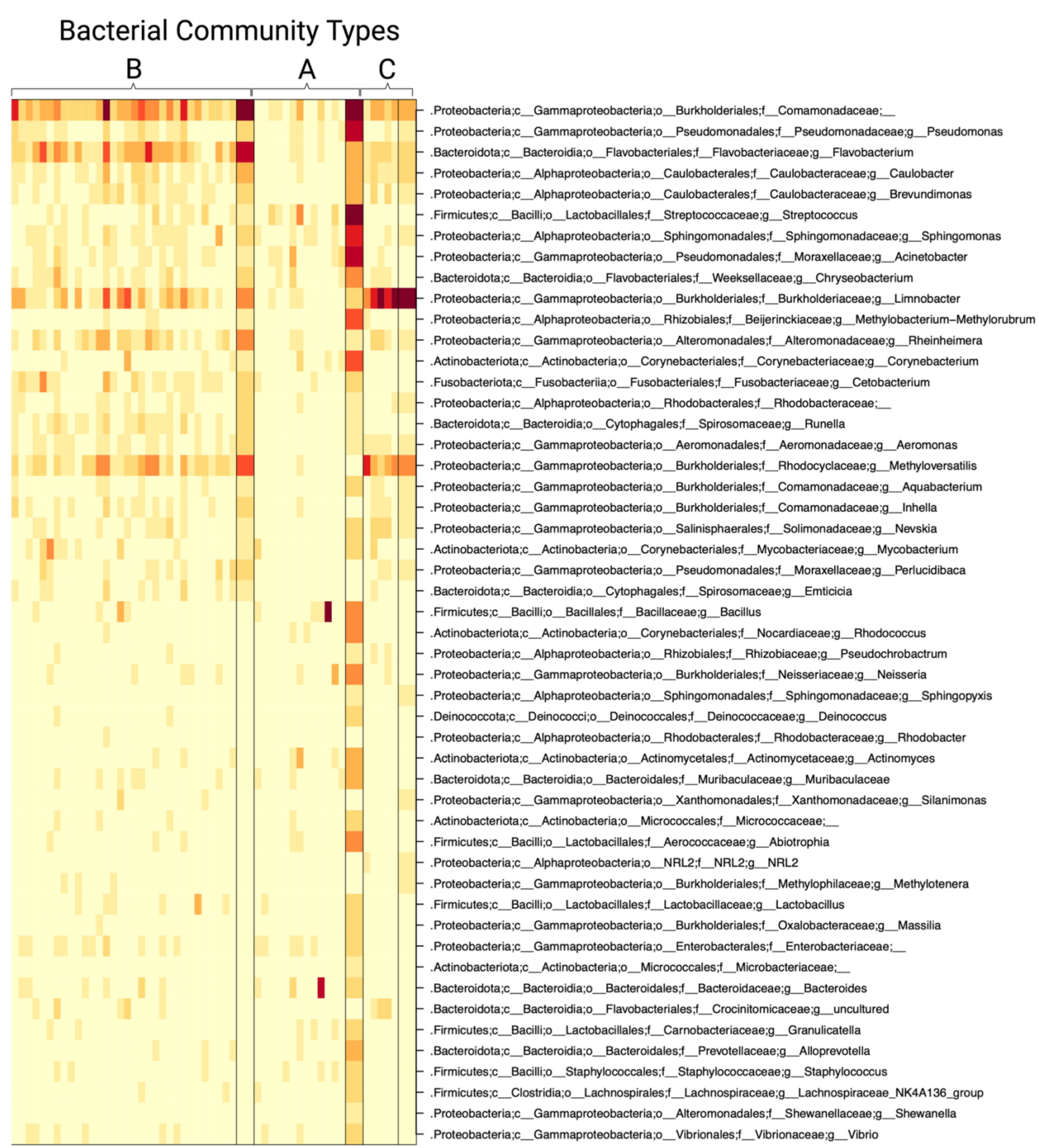

Figure S3. Heat map of the 50 top contributing taxa in the skin microbiome and hierarchical clustering. Heat map showing the skin microbiome data with samples grouped according to the cluster most likely to have generated them. To the right of each cluster the mean relative abundance of the Dirichlet component for that community is shown. Each Narrow columns are individual sample abundances. Darker colors represent increasing relative abundances.

Table S1. Microsatellite and within-group genetic statistics for *P. reticulata* for the three experimental groups and wild-caught source population from 8 previsouly published microsatellite markers: Pret-69, Pret-77 (Watanabe et al., 2005), Pre-9, Pre-15, Pre-18 (Paterson et al., 2005), PP-GATA-5 (Nater et al., 2008), Pr-92 (Becher et al., 2002), Hull 9-1 (Watanabe et al., 2005). Significant differences in allelic richness among populations may indicate variation in the probability of inbreeding. Significant differences in allelic richness among populations are indicative of variation in increases in the probability that inbreeding may occur. Gene diversity measures the mean heterozygosity per individual per population, under inbreeding conditions homozygosity increases but gene diversity is not immediately lost (Charlesworth, 2003). Positive values in F_{IS} can be generated by inbreeding but may also be artificially elevated by technical artifacts such as large allele drop out or null alleles, which can artificially inflate a deficiency of heterozygotes (De Meeûs, 2018; Nei et al., 1975)..

Population	N[a]	A_N[b]	A_R[c]	Ho[d]	Hs[e]	F_{IS}[f]
Inf_Exp Control	42	6.25	6.034	0.419	0.492	0.148
Inf_Exp_Resistant	37	6.75	6.707	0.482	0.507	0.051
Inf_Expe_Susceptible	36	3.87	3.864	0.378	0.423	0.108
Wild Caught	66	12.50	10.507	0.500	0.555	0.100

[a] Number of individuals genotyped
[b] The number of alleles per locus
[c] Allelic richness (based on minimum sample size of 36 individuals)
[d] Observed heterozygosity
[e] Gene diversity
[f] Fixation Index

Table S2. Estimates of Identity disequilibrium (g_2) for the three experimental groups and wild-caught source population. P is the probability that the g_2 statistic is significantly greater than 0 when compared to a random distribution of genotypes. Calculated in Inbreed R (999 permutations). We estimated identity disequilibrium (g_2), the correlation of heterozygosity across loci resulting from inbreeding, which is robust in the presence of null alleles (David et al., 2007). Concurrence among population genetic metrics consistent with patterns inbreeding are needed to determine if phenotypic differences among populations are attributable to inbreeding.

Population	$g_2 \pm$ SE	p
Wild Caught	0.005 ± 0.014	0.3661
Control	-0.031 ± 0.024	0.837
Resistant	0.03 ± 0.037	0.1351
Susceptible	0.05 ± 0.037	0.078

Table S3. Pairwise F_{ST} for the three experimental groups and wild-caught source population. The fixation index (F_{ST}) was assesed to evaluate the degree of population differentiation potentially caused by genetic drift in experimental lines and wild caught source populations

	Control	Resistant	Susceptible	Wild Caught
Control	-			
Resistant	0.02	-		
Susceptible	0.06	0.07	-	
Wild Caught	0.02	0.02	0.03	-
